# Exploring substance use disorder discussions in Native American communities: a retrospective Twitter infodemiology study

**DOI:** 10.1186/s12954-022-00728-z

**Published:** 2022-12-14

**Authors:** Alec J. Calac, Tiana McMann, Mingxiang Cai, Jiawei Li, Raphael Cuomo, Tim K. Mackey

**Affiliations:** 1 UC San Diego School of Medicine, La Jolla, CA USA; 2 UC San Diego Herbert Wertheim School of Public Health and Human Longevity Science, La Jolla, CA USA; 3 Global Health Policy and Data Institute, San Diego, CA USA; 4 Department of Anthropology, UC San Diego, La Jolla, CA USA; 5 S-3 Research, San Diego, CA USA

## Abstract

**Background:**

The opioid epidemic has had a devastating impact on youth from American Indian and Alaska Native (AI/AN) Tribes and Villages, which also experience disparate suicide rates. The use of publicly available social media data originating from AI/AN communities may enhance public health response time to substance use disorder (SUD)-related overdose and augment Tribal public health surveillance systems, but these concepts have yet to be adequately explored. The goal of this exploratory analysis was to identify primary and secondary accounts of overdose and characterize relevant contextual factors in the AI/AN population on social media.

**Methods:**

The Twitter application programming interface was queried for all Tweets containing geocoded data between March 2014 and June 2020 and filtered for the keyword [‘overdose’]. This sample of Tweets (*n* = 146,236) was then restricted to those geolocated from US Tribal lands (*n* = 619). Tweets were manually annotated for primary or secondary accounts of overdose as well as suicidal ideation, substance(s) used, stigma of drug use, and community-wide incidents.

**Results:**

We collected a total of 146,235 tweets that were geocoded and contained the word ‘overdose,’ of which 9.5% were posted on Tribal lands (*n* = 619). 9.4% of these tweets (*n* = 58) met our study inclusion criteria and were mainly posted from Oklahoma (*n* = 26, 45%) and North Carolina (*n* = 13, 22.4%). Most Tweets (*n* = 41, 71%) described a primary account of an overdose and were mostly posted from 2014 to 2015. Less than half of the Tweets (*n* = 27, 46.5%) referenced a specific substance. Those substances mentioned included alcohol, marijuana, methamphetamine, heroin, laundry softener, cocaine, K2-Spice (synthetic cannabinoid), codeine, morphine, Nyquil, and Xanax.

**Discussion:**

Though exploratory, our study identified SUD-related content self-reported by AI/AN communities on Twitter, especially in Oklahoma and North Carolina. These results may assist in the future design and detection of infodemiology trends and early warning signs that can better facilitate intervention specific to the ongoing Tribal opioid epidemic. While all data were collected from the public domain, additional care should be given to individual and community privacy.

## Introduction

Over the last two decades, drug overdose-related mortality has increased more than threefold in the United States (US), with specific mortality rates of different opioid-classes of drugs, such as synthetic opioids and fentanyl, having increased by upwards of 30-fold in the general population [1]. Conditions have continued to worsen with opioid-related deaths now accounting for more than two thirds of all drug-related overdose, leading to a 2017 declaration from then US President Donald J. Trump that the opioid epidemic constituted a national health emergency, leading to the launch of anti-drug advertising campaigns and new government funding opportunities for public health interventions [2–4]. Notably, increasing overdose-related mortality rates have been the most prominent along racial and ethnic lines [5, 6].

Indigenous Peoples in the US, referred to as American Indians and Alaska Natives (AI/AN), experienced the largest percent increase in drug overdose-related mortality compared to other racial and ethnic groups from 1999 to 2015 in non-metropolitan areas (519% increase: 3.8–19.8 per 100,000); yet these figures may be underestimated by up to 35% due to racial misclassification [7]. Concerningly, recent data show that AI/AN drug overdose-related mortality rates are still increasing after surpassing those of the non-Hispanic white population in 2020, meaning that AI/AN have a higher drug overdose mortality burden than any other racial or ethnic group [8–10]. Recognizing the urgency needed around addressing overdose mortality in this population, the US Centers for Disease Control and Prevention (CDC) has pledged its commitment to specifically address shortcomings of opioid-related risk prevention experienced by the 574 federally recognized AI/AN Tribes and Villages in the US.

Despite the disproportionate burden AI/AN communities face from overdose mortality, few culturally responsive substance abuse management and prevention solutions are available that incorporate community values and resilience, and current surveillance systems have issues with AI/AN data quality, accuracy, and timeliness [8]. One approach to augment current surveillance is through the use of infodemiology (i.e., the science of distribution and determinants of information in an electronic medium) [11]. Prior infodemiology studies have found that the use of social media as a medium to generate close-to-real-time public health data, especially within the context of substance use, has the potential to address health disparities and promote health equity goals in minority communities [12–14].

Hence, the goal of this study was to conduct an exploratory retrospective infodemiology study to identify and characterize overdose-related discussions specific to AI/AN communities on the popular microblogging site Twitter. Though data is not readily available on AI/AN use of specific social media platforms, according to the Pew Research Center, Twitter continues to represent a popular platform for user engagement with an estimated 23% of U.S. adults reporting use [15]. Though other platforms—such as Instagram, Facebook, YouTube, and Snapchat—have higher rates of use than Twitter, the platform nevertheless represents a readily accessible and important source of information, particularly in the context of geospecific data that are geolocated or geotagged by users [15]. Concordantly, we are unaware of any infodemiology study that has specifically explored overdose-related experiences and disparities specific to AI/AN communities, despite growing use of these platforms among AI/AN users.

## Methods

### Ethics statement

IRB approval was not required as all data collected in this study was available in the public domain and results from the study have been anonymized and are presented in the aggregate.

### Data collection

The Twitter streaming application programming interface (API), in combination with cloud computing services on Amazon Web Services, was used to continuously collect public Twitter posts from May 2014 to June 2020. Data were then filtered for messages that included geospatial coordinates (latitude and longitude) enabled by users and collected in JSON format, with engagement metrics (e.g., likes, comments), text of the post, and time of the post [16]. In this study, the Twitter streaming API was queried for all Tweets posted within the collection timeframe with the specific keyword filter [‘overdose’]. A total of 146,235 Tweets (Initial Sample) with location metadata were obtained within the geographic borders of the United States. This sample was further filtered for Tweets originating from Tribal lands, defined as an AI/AN Reservation, Alaska Native Village Statistical Area, Trust Land, State Designated Tribal Statistical Area, Tribal Designated Statistical Area, or Oklahoma Tribal Statistical Area using TIGER/Line Shapefiles (latitude and longitude ranges) available from the U.S. Census Bureau (Filtered Sample) using a clipping function available in ArcGIS Desktop (version 10.6) [17]. The boundaries were set based on federally recognized American Indian reservations and off-reservation trust lands through the Census Bureau's Boundary and Annexation Survey of Tribal lands. Queries with other keyword filters returned much smaller unfiltered national samples [‘O.D.’, *n* = 7,039; ‘oodee’, *n* = 11,186] and were excluded from the analysis. While this approach limited the scope of the analysis, it provided adequate content for an initial exploratory analysis of purported overdose conversations as self-reported by users located on Tribal lands.

### Data analysis

The Filtered Sample contained 619 Tweets geolocated and originating from Tribal lands. These Tweets were first reviewed by first and second author AC and TM and manually annotated using an open inductive coding scheme with content describing self-reported accounts of overdose based on prevalent AI/AN behavioral health disparities on Tribal lands (e.g., suicidal ideation, stigma of drug use). All covariates were binary (signal or not signal), enabling further categorization of a Tweet into multiple content categories. Coded content from the Filtered Sample had to be substantive, rather than an individual only tagging an account or predominantly using emojis, ensuring that SUD characteristics of interest described above could be inductively coded/categorized. Annotations were reviewed, and differences were reconciled by all authors until consensus was made. All authors have experience with reviewing and coding social media data involving substance use. A kappa inter-coder reliability score of 0.93 was obtained.

## Results

A total of 619 Tweets from Tribal lands were retrieved from the Twitter API which contained the keyword ‘overdose,’ which were posted between March 2014 and June 2020. Virtually all coded content was posted between April 2014 and October 2015, a period when tweets with geolocation attributes were more readily available due to subsequent changes in Twitter’s privacy policies concerning users’ preferences and setting for geolocation or geotagging of posts. A total of 58 Tweets (9.4%) from 25 Tribal lands met the specified coding and inclusion criteria for content describing accounts of overdose. De-identified and paraphrased example Tweets are provided in Table 1. We also note that we did not observe any Tweets in the dataset that were part of an AI/AN substance use prevention campaign or outreach program.

**Table 1:** Paraphrased and de-identified example Tweets describing overdose from users geolocated Tribal lands between 2014 and 2015

State	Primary account	Secondary account	Substance	SI	Signal (%)	Paraphrased examples	
OK	18 (31.0%)	8 (13.8%)	11 (18.9%)	11 (18.9%)	26 (45%)	Woke up early because my friend overdosed. Why!?	You overdosed in school. Get out of this conversation
						Wouldn’t it be great if I just overdosed on Xanax	I’d rather overdose than live
NC	10 (17.2%)	3 (5.1%)	8 (13.8%)	8 (13.8%)	13 (22%)	I hope I overdose, bye all	Way too many community overdoses from K2/Spice in the last month
CA	4 (7.0%)	1 (1.7%)	3 (5.1%)	3 (5.1%)	5 (8.6%)	Hand me the pills to OD	Our teens are dying from methamphetamine
NE	2 (3.5%)	–	1 (1.7%)	–	2 (3.5%)	I’ve OD and died in the past	Took too much [laundry] softener this time. OD’d

OD = Overdose, K2 = synthetic cannabinoid, SI = suicidal ideation

Iterative themes that emerged during the coding phase included primary and secondary accounts of overdose, suicidal ideation, stigma of drug use, stigma origin (peer, community, school-based setting), mention of specified substances (e.g., alcohol, methamphetamine, Xanax), and community-wide incidents (e.g., cluster events, polysubstance use, synthetic drug use). From coded content describing overdose, 41 (71%) described a primary account of an overdose and 17 (29%) described a secondary account of an overdose. A total of 27 Tweets (46.5%) referenced specific substances, sometimes in co-use, which included: alcohol, marijuana, methamphetamine, heroin, laundry softener, cocaine, K2 (synthetic cannabinoid), codeine, morphine, Nyquil, and Xanax. A total of 3 Tweets (5.2%) included emojis depicting cigarettes, pills and syringes (drug paraphernalia), skulls, and heavenly angels. While we were unable to ascertain the age of Twitter users posting from within AI/AN communities, we did observe posts that referenced place-based contexts such as schools and gatherings of friends that require further study in relation to understanding the impact of this crisis among AI/AN youth and young adults.

The majority of the coded dataset originated from Tribal lands in Oklahoma (26 Tweets), followed by Tribal lands in North Carolina (13 Tweets), California (5 Tweets), and Alaska (3 Tweets). There were 2 Tweets each from Tribal lands in Nebraska, Alabama, and Washington, and single Tweets from Tribal lands in Michigan, New Jersey, Louisiana, Minnesota, and Virginia. Overall, Tweets were detected in a total of 12 states. Across all states, 6 Tribal lands in Oklahoma and 1 Tribal land in North Carolina accounted for 48% and 21% of the coded Tweets, respectively.

### Limitations

This study has some limitations. Data was collected from the public streaming Twitter API limited to geolocated posts during a prescribed time period (2014–2020). This was done to maximize the potential to detect geolocated posts from a specific AI/AN geographic boundary or shapefile. This limits any generalizability of results, as other sources of data (e.g., inclusion of additional platforms, use of Twitter firehose data, and including non-geolocated posts), may have yielded additional data relevant to the study aims though may not have included geo-sensitive data specific to AI/AN communities of interest. Hence, results from this study are not representative of substance use in the specific AI/AN communities we identified. It is a convenience sample of individual users who had access to the Internet, publicly posted behavior-related content on a single social media platform and had location metadata. Further, all coded results were from 2014 to 2015, a relatively short period of time and lags behind reporting results from this study.

We also acknowledge that it is impossible to confirm the accuracy and validity of the content we identified as it is not linked to databases managed by local and state public health departments and Tribal Epidemiology Centers. Importantly, attention must also be dedicated to individual and community privacy in the conduct of this type of research. Finally, because this was an exploratory analysis with only one keyword (i.e., “overdose”), this may have limited the content we detected, due to the potential use of pseudonyms and slang terms for overdose and other associated behaviors and limits on data queries. Future analyses should broaden queries using region and group-specific keywords that relate to substance use and overdose in minority populations and more expansive data-collection methodologies that include additional platforms popular among AI/AN communities.

## Discussion

The opioid epidemic has coincided with the increasing use of social media platforms such as Twitter to discuss SUD-related topics, including first and second-hand experiences with overdose [18]. Our study was able to identify and characterize 58 tweets specifically posted from Tribal lands from 2014 to 2015, where users actively discuss struggles with SUD, overdose, and mental health challenges. Most coded posts (70.6%) described a primary account of an overdose, also annotated for co-occurring substance use or suicidal intent, suggesting that Twitter may be a useful platform to understand drivers of SUD in this population.

Substance use content was detected from over two dozen AI/AN communities; concerningly, almost half of this content originated from Tribal lands in Oklahoma. Tribes in Oklahoma, along with others across the country, have actively litigated against the pharmaceutical industry for their role in the spread of opioid-related health burden across Tribal lands. The Cherokee Nation, one of the first Tribes to sue the pharmaceutical industry over the opioid epidemic, reported in legislation that “there were so many opioids within the Tribe's 14-county reservation in 2015 that it amounted to 107 pills for every adult resident. [19]” In 2022, hundreds of AI/AN Tribes reached a settlement on National Prescription Opiate Multi-District Litigation, leading to more than half a billion dollars in funds that will be used to address AI/AN opioid addiction and long-term treatment.

This study represents a first step in determining whether social media data can be used to explore SUD health-related disparities among the AI/AN population living on Tribal lands. Unlike other social media platforms, Twitter generally provides the most robust location metadata (i.e., latitude and longitude). However, only a small percent of Tweets have available local metadata, having the potential to underrepresent the magnitude of a particular health topic-of-interest. While previous Twitter research has explored engagement with AI/AN-focused hashtags and topics relevant to AI/AN youth, this study explicitly limited data collection to areas where virtually every resident is AI/AN, increasing the sensitivity of our data collection [20].

Increasing the responsiveness and sensitivity of traditional mortality-based surveillance systems, which only capture limited dimensions of data (e.g., case rates, mortality rates), may help address the national substance use emergency faced by metropolitan and non-metropolitan-residing AI/ANs and other minorities in the US. Future research should use other platforms, explore other health topics, and consider comparing social media-based data to relevant electronic health record systems, such as large-scale data warehouses maintained by Regional Health Information Organizations, and other region-specific databases (e.g., behavioral risk factor surveillance surveys), including those maintained by local and state public health departments, Tribes, and Tribal Epidemiology Centers.

Additionally, attention must be dedicated to individual and community privacy in the conduct of this type of research due to known stigma associated with substance use and the potential that disparities evident in one AI/AN community may be erroneously or maliciously generalized to produce stereotypes about the entire AI/AN population [21]. To address this in our study, displayed Tweets were paraphrased and identifying metadata and account mentions were removed. We present paraphrased Tweets and an example of the inductive coding scheme we applied to the 58 Tweets. Therefore, risk of re-identification by public users is minimal.

Overall, study results may also be of interest to community advocates, Tribal leaders, and public health researchers interested in addressing the growing burden of substance use in the AI/AN population, especially as it pertains to AI/ANs residing in non-metropolitan areas such as Tribal lands. Results presented in this study stem from an exploratory analysis and should be further validated with confirmatory community-based participatory approaches and other data sources (e.g., surveys, focus groups). Further, this infodemiology approach, which may have the potential to be used at scale with residents on Tribal lands, may represent a useful adjunct for existing Tribal public health surveillance systems seeking to detect emerging Tribal public health challenges in near real-time using techniques such as supervised machine learning.

For example, our analysis discovered a tweet from Nebraska Tribal lands reporting increased use of K2-Spice (synthetic cannabinoid) in the community. De-identified information generated from these results can be routed to the proper public health authorities and acted upon to protect the public health. In addition, the development of comparative infodemiology studies that examine different demographic groups may reveal between-group differences and behaviors that can inform the development of policies and programs addressing minority substance use for specific and distinct communities.

In conclusion, overdoses resulting from substance use in AI/AN and other predominantly minority-residing communities may be largely preventable if actionable social media data is responsibly collected and results made available to key stakeholders. Depending on the scope of data collection and reasonable expectations for user privacy, this may require data-sharing agreements between social media platforms, public health authorities, and researchers, ensuring data is only accessed to protect communities at heightened risk of substance use and other harms.

## References

[1] Singh GK, Kim IE, Girmay M (2019). Opioid epidemic in the United States: empirical trends, and a literature review of social determinants and epidemiological, pain management, and treatment patterns. Int J MCH AIDS.

[2] Hedegaard H, Miniño AM, Warner M (2018). Drug overdose deaths in the United States, 1999–2017. NCHS Data Brief.

[3] Scholl L, Seth P, Kariisa M, Wilson N, Baldwin G (2018). Drug and opioid-involved overdose deaths-United States, 2013–2017. MMWR Morb Mortal Wkly Rep.

[4] Opioid Crisis: Status of Public Health Emergency Authorities. *Government Accountability Office.* Published September 26, 2018. Accessed May 27, 2022. https://www.gao.gov/products/gao-18-685r

[5] Wilson N, Kariisa M, Seth P, Smith H, Davis NL (2020). Drug and opioid-involved overdose deaths-United States, 2017–2018. MMWR Morb Mortal Wkly Rep.

[6] Larochelle MR (2021). Disparities in opioid overdose death trends by race/ethnicity, 2018–2019, from the HEALing communities study. Am J Public Health.

[7] Mack KA, Jones CM, Ballesteros MF (2017). Illicit drug use, illicit drug use disorders, and drug overdose deaths in metropolitan and nonmetropolitan areas—United States. MMWR Surveill Summ.

[8] Opioid Overdose Prevention in Tribal Communities. CDC/National Center for Injury Prevention and Control. Reviewed May 5, 2021. Accessed May 3, 2022.

[9] Friedman JR, Hansen H (2022). Evaluation of increases in drug overdose mortality rates in the US by race and ethnicity before and during the COVID-19 pandemic. JAMA Psychiat.

[10] Mpofu E, Ingman S, Matthews-Juarez P, Rivera-Torres S, Juarez PD (2021). Trending the evidence on opioid use disorder (OUD) continuum of care among rural American Indian/Alaskan Native (AI/AN) tribes: a systematic scoping review. Addict Behav.

[11] Eysenbach G (2009). Infodemiology and infoveillance: framework for an emerging set of public health informatics methods to analyze search, communication and publication behavior on the Internet. J Med Internet Res.

[12] Nasralah T, El-Gayar O, Wang Y (2020). Social media text mining framework for drug abuse: development and validation study with an opioid crisis case analysis. J Med Internet Res.

[13] Hu H, Phan N, Chun SA (2019). An insight analysis and detection of drug-abuse risk behavior on Twitter with self-taught deep learning. Comput Soc Netw.

[14] Kalyanam J, Katsuki T, Lanckriet RG, Mackey TK (2017). Exploring trends of nonmedical use of prescription drugs and polydrug abuse in the Twittersphere using unsupervised machine learning. Addict Behav.

[15] Social Media Fact Sheet. Pew Research Center. Published April 7, 2021. Accessed August 31, 2022. https://www.pewresearch.org/internet/fact-sheet/social-media/?menuItem=b14b718d-7ab6-46f4-b447-0abd510f4180

[16] Twitter Developer. Documentation: Twitter API. Accessed May 5, 2022. https://developer.twitter.com/en/docs

[17] TIGER/Line Shapefile, 2018, nation, U.S., Current American Indian/Alaska Native/Native Hawaiian Areas National (AIANNH) National. U.S. Census Bureau. Last Updated February 24, 2021. Accessed May 5, 2022. https://catalog.data.gov/dataset/tiger-line-shapefile-2018-nation-u-s-current-american-indian-alaska-native-native-hawaiian-area

[18] Yeung AWK, Kletecka-Pulker M, Eibensteiner F (2021). Implications of Twitter in health-related research: a landscape analysis of the scientific literature. Front Public Health.

[19] Young, M. Native American tribes in Oklahoma, U.S. reach $590 million opioid settlement. *The Oklahoman*. Published February 2, 2022. Accessed August 31, 2022. https://www.oklahoman.com/story/news/2022/02/02/cardinal-health-opioid-settlement-mckesson-johnson-johnson-oklahoma-tribes/9301749002/

[20] Around Him, D., et al. Twitter Analysis Can Help Practitioners, Policymakers, and Researchers Better Understand Topics Relevant to American Indian/Alaska Native Youth. *Child Trends*. Published November 17, 2020. Accessed August 28, 2022. https://www.childtrends.org/publications/twitter-analysis-practitioners-policymakers-researchers-understand-topics-american-indian-alaska-native-youth

[21] Williams ML, Burnap P, Sloan L (2017). Towards an ethical framework for publishing twitter data in social research: taking into account users’ views, online context and algorithmic estimation. Sociology.

